# RNA Binding Proteins as Potential Therapeutic Targets in Colorectal Cancer

**DOI:** 10.3390/cancers16203502

**Published:** 2024-10-16

**Authors:** Vikash Singh, Amandeep Singh, Alvin John Liu, Serge Y. Fuchs, Arun K. Sharma, Vladimir S. Spiegelman

**Affiliations:** 1 Division of Pediatric Hematology and Oncology, Department of Pediatrics, The Pennsylvania State University College of Medicine, Hershey, PA 17033, USA; vsingh2@pennstatehealth.psu.edu (V.S.);; 2 Department of Pharmacology, Penn State Cancer Institute, The Pennsylvania State University College of Medicine, Hershey, PA 17033, USA; asingh8@pennstatehealth.psu.edu (A.S.); asharma1@pennstatehealth.psu.edu (A.K.S.); 3 Department of Biomedical Sciences, School of Veterinary Medicine, University of Pennsylvania, Philadelphia, PA 19104, USA; syfuchs@vet.upenn.edu

**Keywords:** RNA-binding protein (RBP), colorectal cancer (CRC), small molecule inhibitor, LIN28, IGF2BP, HuR, Musashi, CELF1

## Abstract

RNA-binding proteins (RBPs) play critical roles in regulating post-transcriptional gene expression, managing processes such as mRNA splicing, stability, and translation. In normal intestine, RBPs maintain the tissue homeostasis, but when dysregulated, they can drive colorectal cancer (CRC) development and progression. Understanding the molecular mechanisms behind CRC is vital for developing novel therapeutic strategies, and RBPs are emerging as key players in this area. This review highlights the roles of several RBPs, including LIN28, IGF2BP1–3, Musashi, HuR, and CELF1, in CRC. These RBPs regulate key oncogenes and tumor suppressor genes by influencing mRNA stability and translation. While targeting RBPs poses challenges due to their complex interactions with mRNAs, recent advances in drug discovery have identified small molecule inhibitors that disrupt these interactions. These inhibitors, which target LIN28, IGF2BPs, Musashi, CELF1, and HuR, have shown promising results in preclinical studies. Their ability to modulate RBP activity presents a new therapeutic avenue for treating CRC. In conclusion, RBPs offer significant potential as therapeutic targets in CRC. Although technical challenges remain, ongoing research into the molecular mechanisms of RBPs and the development of selective, potent, and bioavailable inhibitors should lead to more effective treatments and improved outcomes in CRC.

## Simple Summary

RNA-binding proteins (RBPs) are crucial in regulating gene expression in the intestine, and their abnormal activity can lead to colorectal cancer (CRC). This review explores how specific RBPs, such as LIN28, IGF2BPs, Musashi, HuR, and CELF1, contribute to CRC by affecting the stability and translation of key cancer-related genes. These proteins can promote tumor growth, spread, and resistance to treatments. Although targeting RBPs is challenging, recently discovered small molecules can disrupt the harmful interactions, showing promise for new CRC therapies. Further research and development of RBP-targeting agents should lead to better treatment options for CRC patients.

## 1. Introduction

The regulation of eukaryotic genes occurs at both the transcriptional and post-transcriptional levels, resulting in unique expression profiles tailored to specific conditions [1,2]. Global steady-state mRNA and protein measurements show modest correlations, which have been taken as evidence that mRNA levels account for approximately 40% of the variation in protein levels, indicating dominating post-transcriptional effects [3]. Following transcription, mRNA undergoes various processing steps, primarily managed by RBPs [4]. Previously, 1542 protein-coding genes have been designated RBPs, comprising around 7.5% of all human protein-coding genes [5]. Later, another study updated the list and reported the 1914 RBPs in humans [6]. RBPs are involved in post-transcriptional gene regulation by 5’capping, mRNA stability, transport, splicing, and translation [5]. RBPs interact with mRNA molecules, forming ribonucleoprotein (RNP) complexes whose composition dynamically changes during RNA processing. While RBPs can bind to different RNA classes, including ribosomal RNAs, snRNAs, and non-coding RNAs, approximately half of RBPs primarily function by binding to mRNAs, exerting specific regulatory roles [7].

The dysregulation of RBP expression can lead to diseases like cancer because it plays a role in critical biological processes such as transcription, translation, mRNA stability, and transport [8,9,10]. Ample research has highlighted how changes in RBP expression, localization, or post-translational modifications contribute to tumorigenesis by upregulating oncogenes and downregulating tumor suppressor genes. Consequently, there is growing interest in illustrating the functions of RBPs in cancer and exploring their potential as targets for therapeutic interventions. Colorectal cancer (CRC) is one of the many disorders that can result from significant changes or disruptions in these RBPs.

Worldwide, CRC is the second most common cause of cancer-related fatalities as well as the third most common cancer diagnosis [11]. Effective chemopreventive and chemotherapeutic techniques are urgently needed, as colorectal cancer continues to be a major cause of cancer mortality despite significant advances in its scientific understanding and treatment [12]. Even with treatments including chemotherapy, immunotherapy, and surgery, the 5-year survival rate for individuals with distant metastases in colorectal cancer is only 14%, a sharp decline from the 90% rate for those with localized disease [13,14]. The development of CRC proceeds stepwise, starting with normal mucosa and ending with adenomatous polyps and carcinoma. The accumulation of genetic changes that give tumor cells a selective growth advantage is an outcome of the multistage process [15]. These genetic alterations disrupt the equilibrium between cell division and apoptosis, which affects oncogenic and tumor-suppressive signaling pathways. Besides genetic alterations, RNA-binding proteins (RBPs) are pivotal in post-transcriptional regulation. It has been established that RBP dysregulation or abnormal activity contributes to tumorigenesis and plays major roles in the development and progression of cancer by influencing the stability and translation of mRNAs that encode for tumor suppressors and oncogenes [16,17]. Understanding the function of these proteins and their potential targeting could provide new opportunities for CRC treatment.

Despite numerous attempts to develop therapeutic strategies against RBPs, achieving success has proven challenging, primarily due to the complexities involved in directly targeting RBPs or the selectivity of interacting mRNAs. In this context, RBPs reported in the published literature regarding CRC include LIN28, IGF2BP1, Musashi (MSI), CELF1, and HUR. These RBPs are essential regulators in controlling gene expression post-transcriptionally. Its significant role in modulating target mRNA expression influences cellular growth, proliferation, differentiation, and metabolism. Dysregulation of gene expression has been associated with various diseases, including cancer, where its overexpression is linked to increased tumor growth, metastasis, and therapy resistance. RBP stabilizes the mRNA of oncogenes and growth-promoting factors in cancer cells, contributing to the malignant phenotype. Consequently, targeting these RBPs and their downstream effectors has been identified as a viable therapeutic option for treating cancer.

In light of these challenges, various high-throughput systems have been developed to screen for small molecule inhibitors, utilizing techniques such as fluorescence resonance energy transfer (FRET), bioluminescence resonance energy transfer (BRET), and fluorescence polarization (FP). These methods are particularly valuable for measuring molecular distances, essential for analyzing protein-RNA interactions and identifying potential therapeutic modulators like LIN28-let-7 microRNA inhibitors, which are emerging as promising anticancer agents. Additionally, fluorescence polarization and intensity-based assays have effectively identified therapeutic agents for diseases like Alzheimer’s by detecting changes in fluorescence upon molecular binding. Advanced techniques like AlphaScreen, which uses biotin-modified RNAs and bead-linked binding partners, help in detecting protein–RNA interactions, such as those involving EZH2 and noncoding RNAs. Similarly, the catalytic enzyme-linked click chemistry assay (cat-ELCCA) enhances small-molecule screening capabilities by generating chemiluminescent signals from bioorthogonal handles on RNAs upon binding. These sophisticated methodologies collectively offer a comprehensive toolkit for probing molecular interactions and accelerating the development of targeted therapies [18].

## 2. Lin28

### 2.1. (Patho)physiological Function

LIN28, an evolutionarily conserved RBP, was first identified in Caenorhabditis elegans for its developmental roles [19]. In mammals, two paralogs, LIN28A and LIN28B, share similar structures and functions. They play crucial roles in various physiological processes, including tissue development, cellular growth, metabolism, and maintaining the capabilities for pluripotency and reprogramming across human and animal systems [20,21]. These proteins not only regulate growth by interacting with mRNAs of essential ribosomal and cell cycle proteins like RPS13, Cyclins A, B, D, CDK2, CDK4, and CDC25A, enhancing their translation and thus controlling cellular proliferation but also significantly influence the maturation of the microRNA let-7, a critical factor in developmental timing and tumor suppression [22]. LIN28A and LIN28B disrupt let-7 maturation by binding to its precursors through their cold shock domain (CSD) and zinc knuckle domain (ZKD), effectively blocking the cleavage steps that convert pre-miRNA into mature miRNA. This dual function highlights their pivotal role in both promoting growth and inhibiting the maturation of miRNAs critical for cell regulation, as evidenced by experiments showing that mice genetically engineered to overexpress LIN28A are larger, whereas those lacking it display dwarfism, underscoring LIN28A’s key influence on growth and development [23]. LIN28A maintains embryonic stem cell pluripotency and enhances epigenetic reprogramming through OCT4/SOX2/KLF4 [24]. LIN28B also plays a role in pluripotency [25], but its involvement in cancer has been more extensively investigated.

Elevated expression of LIN28A or LIN28B in the context of activated Wnt signaling was shown to be associated with invasive intestinal adenocarcinoma in both murine models and human clinical samples [26]. Thus, the study demonstrated that LIN28 overexpression, when paired with mutations in the Wnt pathway, particularly β-catenin, enhances mouse tumor formation, proliferation, and invasiveness, and conversely, suppressing LIN28 expression in these models led to reduced tumor volume and increased differentiation, highlighting its potential as a therapeutic target. In CRC, LIN28B is overexpressed in 30–60% of patients, and is correlated with more aggressive and invasive tumor characteristics and linked to poor prognosis [26,27,28]. Another set of studies demonstrated that LIN28B acts as an oncogene in a genetic mouse model of CRC and promotes liver metastasis in a subcutaneous xenograft model [26,27,28,29]. LIN28B suppresses the biogenesis of the let-7 family microRNA and reduced let-7 expression has been observed in numerous human cancers. Disruption of let-7 expression is linked to poor prognosis and an increase in metastasis in various cancers, including colorectal, ovarian, lung, and breast cancer. Several studies have investigated the role of the let-7 and LIN28B axis in cancer progression [30]. LIN28B was also shown to enhance cell migration and invasion by upregulating CLDN1, a tight junction protein, and activating NOTCH3 signaling. The findings suggest that targeting the LIN28B/CLDN1/NOTCH3 axis could provide new therapeutic strategies for metastatic CRC. The study also shows that pharmacologic inhibition of NOTCH3 reduces LIN28B-induced liver metastasis, offering potential clinical applications for managing CRC metastasis. These studies underscore the critical role of LIN28 in the progression of CRC and suggest that targeting it with small molecule inhibitors (Table 1) could be beneficial in treating aggressive forms of this cancer [31].

### 2.2. Inhibitors

One of the first research studies reports the development of a fluorescence polarization-based high-throughput screening assay to identify inhibitors of the Lin28-pre-let-7g interaction, a crucial pathway implicated in tumorigenesis and stem cell renewal. In this study, a library of 2768 pharmacologically active compounds were screened, including FDA-approved drugs, and identified several small molecule inhibitors that disrupt this interaction. Two compounds, in particular, 6-hydroxy-DL-DOPA and SB/ZW/0065 were shown to restore the processing of pre-let-7g in the presence of Lin28, validating the assay and highlighting the therapeutic potential of targeting this pathway [32]. A separate study utilized a high-throughput screening of 16,000 drug-like molecules, resulting in the identification of a compound, N-methyl-N-[3-(3-methyl [1,2,4]triazolo [4,3-b]-pyridazine-6-yl)phenyl]acetamide, referred to as 1632. This inhibitor disrupts the interaction between Lin28 and let-7, effectively promoting the maturation of let-7 in Lin28-expressing cancer cells and inducing differentiation in mouse embryonic stem cells. The compound also decreased tumor-sphere formation in cancer cell lines, showcasing its potential as a therapeutic agent targeting the Lin28/let-7 axis in diseases characterized by deregulated stem cell pathways and cancer progression [33]. Another study reports on developing a novel fluorescence resonance energy transfer (FRET)-based high-throughput screening system designed to identify small-molecule inhibitors of the Lin28-let-7 interaction. Employing bioorthogonal chemistry for precise fluorescent labeling of Lin28, the researchers screened a library of 4500 drug-like compounds. They identified a potent inhibitor, Compound 1, a benzopyranylpyrazole-based molecule, which effectively disrupted the Lin28−let-7 interaction [34]. An exciting study outlines the development of a fluorescence polarization assay (FPA) to identify small-molecule inhibitors targeting both domains of LIN28. Through high-throughput screening of 101,017 compounds, six were found to inhibit LIN28 binding and disrupt LIN28-mediated let-7 oligouridylation effectively. Studies show that the zinc-knuckle domain (ZKD) of LIN28 is an essential player in the regulation of let-7 and contains a cavity that small molecules can target. Notably, TPEN was shown to destabilize the ZKD, while LI71 modulated the cold shock domain (CSD) of LIN28. Additionally, a minimal scaffold, MNA (5-(Methylamino) nicotinic acid), was identified as a LIN28 inhibitor targeting the cold shock domain, offering a potential new approach for therapeutic intervention in LIN28-driven diseases [35].

Another approach by Lorenz et al. focused on enhancing the catalytic enzyme-linked click chemistry assay (cat-ELCCA) for discovering the inhibitors targeting interaction between pre-let-7 and Lin28. The cat-ELCCA method for the high-throughput screening (HTS) of 127,007 compounds revealed new chemical entities that can inhibit the RNA-binding protein Lin28. Two notable inhibitors identified were N,N′-(1,2-phenylene)-dibenzenesulfonamide derivatives CCG-233094 and CCG-234459. These compounds showed potential by inhibiting the pre-let-7–Lin28 interaction with IC_50_ values of 8.3 and 10.3 μM, respectively. The study demonstrates the efficacy of cat-ELCCA in screening for inhibitors of RNA–protein interactions, offering new avenues for therapeutic development against cancer [36]. Using an FPA-based screening, a separate study identified trisubstituted pyrrolinones that disrupt this interaction. The most effective compound identified was C902, which showed dose-dependent inhibition in electrophoretic mobility shift assays and enhanced the thermal stability of LIN28’s cold shock domain.

Furthermore, C902 increased the mature Let-7 levels in JAR cells, which suggests its potential as a therapeutic agent [37]. A comprehensive in silico screening of 18 million compounds from the ZINC20 library identified 15 potential Lin28 inhibitors. The three lead compounds, namely Ln7, Ln15, and Ln115, were found to specifically target the zinc knuckle domain of Lin28A and Lin28B, effectively blocking the interaction with the Let-7 microRNA. These inhibitors were shown to restore Let-7 expression, suppress oncogenes like SOX2, and exhibit strong inhibitory effects on cancer cell stem-like phenotypes, all while demonstrating minimal impact on Lin28-negative cells, emphasizing their on-target efficacy [38]. Another study later discussed the development of bifunctional small-molecule inhibitors targeting the protein–RNA interaction between LIN28 and let-7. The research uses a virtual alanine scan and structure–activity relationship analysis to identify hotspots for effective binding on LIN28 and synthesizes bifunctional conjugates combining chromenopyrazole with peptides. The key compound identified, PH-223 (also referred to as compound 83), demonstrated significant inhibition of the LIN28–let-7 interaction, offering a new approach to modulate this pathway for potential therapeutic benefits in oncology. This study exemplifies the utility of integrating peptide elements with small molecules to target complex protein–RNA interactions, potentially leading to novel cancer treatments [39]. In a novel approach to finding inhibitors against LIN28, live cell screening was implied where authors used an RNA interaction with a protein-mediated complementation assay (RiPCA) for HTS to identify inhibitors of the Lin28A and pre-let-7d interaction. The study effectively screened 17,797 compounds and highlighted SID-415260 as a standout inhibitor that disrupts this critical RNA–protein interaction. SID-415260 significantly increased mature let-7 levels in Lin28A-expressing cancer cells, demonstrating its potential therapeutic impact. With IC_50_ values of 10.7 μM, 7.4 μM, and 12.5 μM for various let-7 family members, SID-415260 shows promise as a broad-spectrum inhibitor targeting the Lin28–let-7 axis in cancer therapy [40].

**Table 1:** Lin28 inhibitors.

Structure	Name	IC_50_ (µM)	Reference
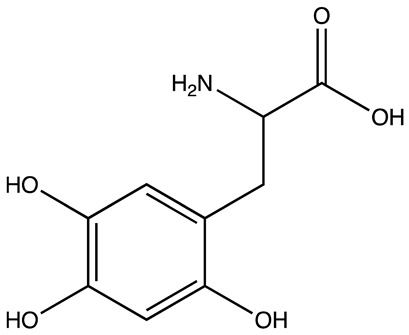	6-Hydroxy-DL-DOPA	7.05	[32]
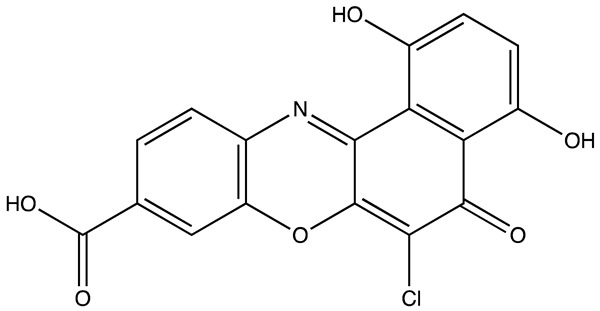	SB/ZW/0065	4.71	[32]
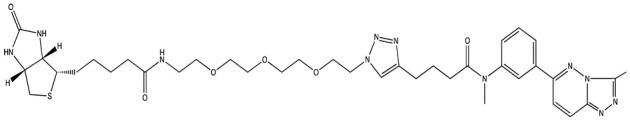	C-1632	8	[33]
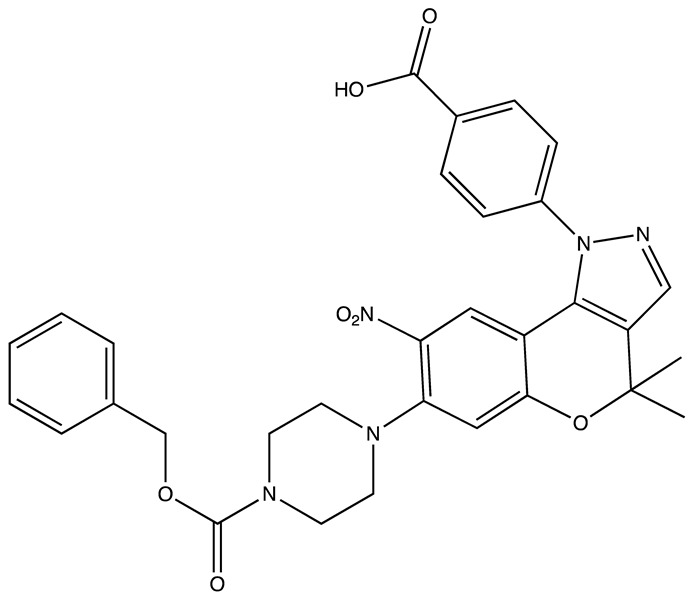	1	4.03	[34]
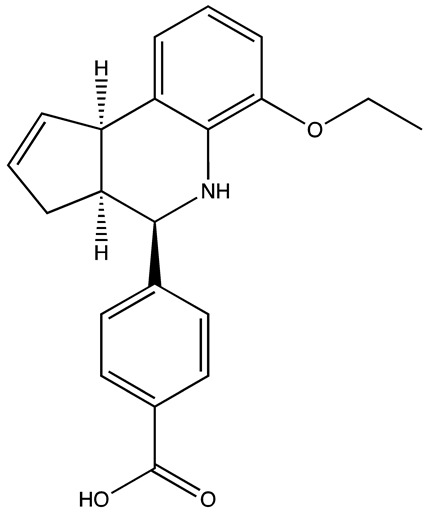	LI71	7	[35]
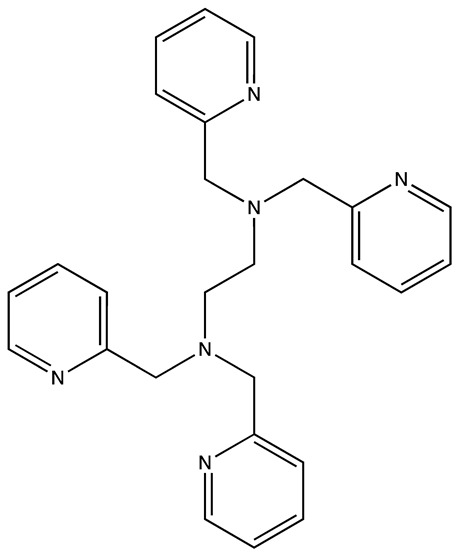	TPEN (LI38)	2.5	[35]
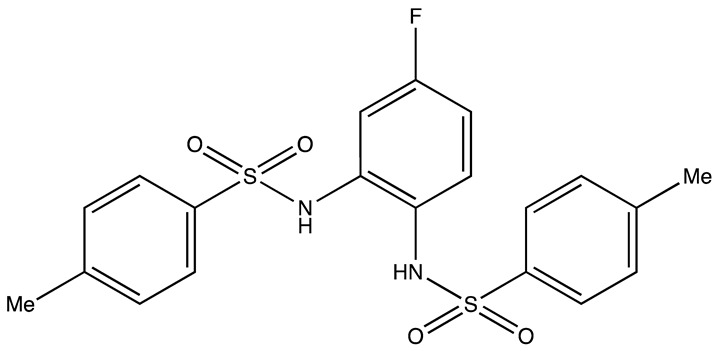	CCG-233094	15.5	[36]
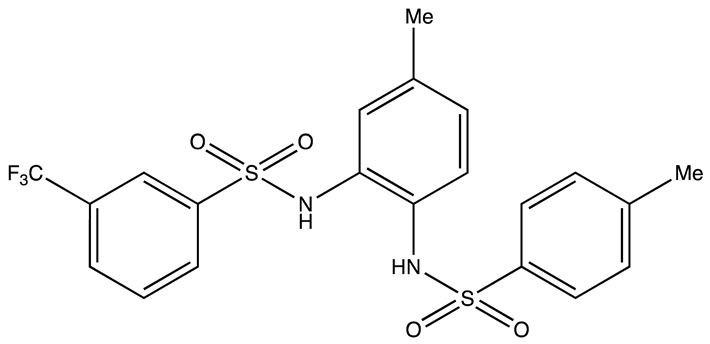	CCG-234459	14.8	[36]
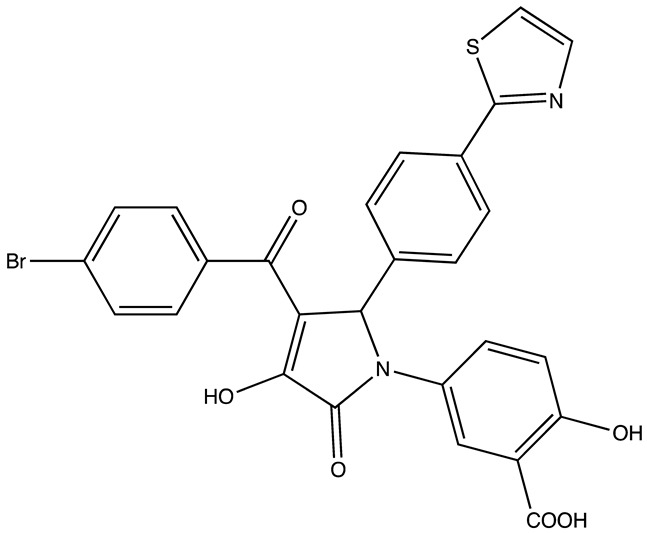	C902	5	[37]
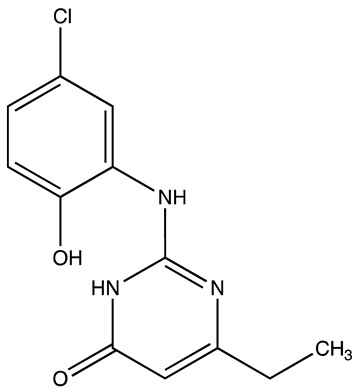	Ln7	45	[38]
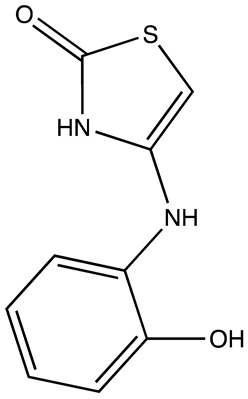	Ln15	9	[38]
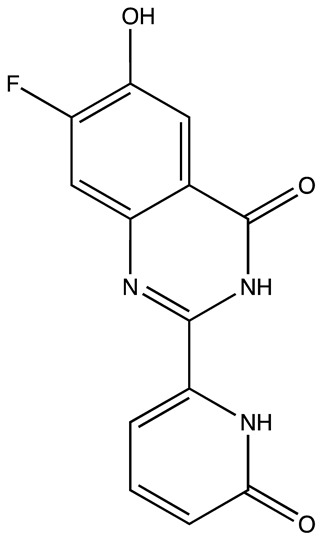	Ln115	21	[38]
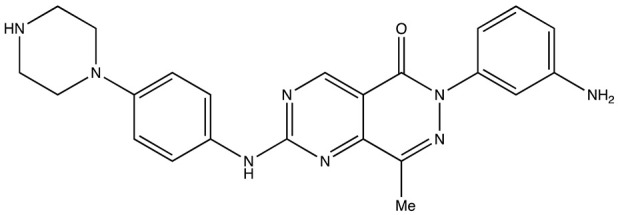	SID-415260	7.6	[40]

**Figure 1:**
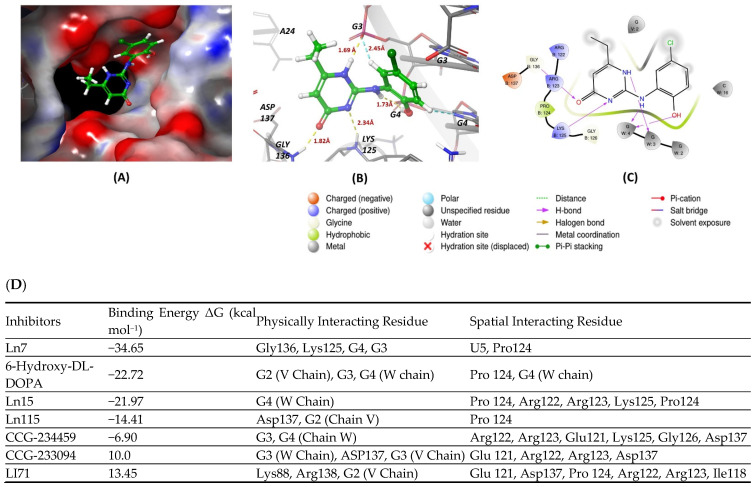
(**A**,**B**) show 3D representations of the binding and surface view of inhibitor Ln7 with RNA binding protein Ln28 (PDBID: 5UDZ). (**C**) shows 2D representations of binding interactions of Ln7. (**D**) The binding energy of inhibitors with RNA binding protein Ln28 along with interacting residues. Color code: green = carbon, white = hydrogen, blue = nitrogen, and red = oxygen.

This review article uses molecular docking to demonstrate the interactions between known inhibitors and RNA-binding proteins (RBPs). This computational method is essential for identifying binding affinities and specific interactions at the amino acid level, thereby predicting the potential efficacy of inhibitors in disrupting RBP–RNA interactions. Such insights are crucial for developing therapeutic strategies against diseases where RBPs play a regulatory role. By visualizing how these inhibitors occupy the binding sites of RBPs, molecular docking provides a predictive framework that guides subsequent experimental validation in optimizing these molecules for increased specificity and effectiveness in drug development processes. All known Lin28 inhibitors were docked to the human LIN28A protein complexed with let-7f-1 microRNA pre-element using PDB ID: 5UDZ to see the binding affinity. The protein binding sites were identified using the Sitemap module of Maestro. The top-ranked site was selected to generate a receptor grid [41]. Docking was performed using Glide and MM-GBSA Prime in Maestro software (version 13.2.128; Schrödinger). The top poses with the lowest binding energies were analyzed, and the binding energies of the compounds are summarized in Figure 1D. Inhibitor Ln7 exhibited more interactions with the protein and RNA and displayed lower binding energy than other inhibitors (Figure 1A–C). A detailed inspection of Ln7’s interactions revealed that it forms hydrogen bonds with both the protein and RNA. The heterocyclic ring of the molecule forms hydrogen bonds via its nitrogen and N-H atoms with lysine125 of the protein and G3 residue of the W chain of RNA, with bond lengths of 2.34 Å and 1.69 Å, respectively. Additionally, the carbonyl group of the ring interacts with glycine136 of the protein via a hydrogen bond with a bond length of 1.82 Å. Moreover, the G4 residue of the W chain in RNA interacts with the inhibitor through hydrogen bonds formed by the benzene ring’s bridged N-H and hydroxy groups, with bond lengths of 1.73 Å and 1.56 Å, respectively. The inhibitor also displayed a weak aromatic hydrogen bond interaction with the G3 residue of RNA. Furthermore, the surface view of the docking pose revealed that Ln28 occupies the protein’s binding site. Although molecular docking serves as a crucial tool for screening inhibitors to disrupt RBP–RNA interactions but it encounters significant challenges also. Primarily, it relies on static models that fail to capture the dynamic behaviors of RBPs and RNA, potentially missing essential conformational changes during binding. Additionally, these simulations might not explore the full conformational space, often neglect the impact of water molecules and ions, and typically overlook RNA’s post-transcriptional modifications, all vital for precise RBP–RNA interaction modeling. Moreover, most docking algorithms are primarily designed for protein–protein interactions and may not be completely suitable for the distinct molecular interactions specific to RBP–RNA binding. This inadequacy signals a pressing need for more sophisticated or hybrid computational strategies to improve the accuracy of predictions.

## 3. IGF2BP’s

### 3.1. (Patho)physiological Function

Insulin-like growth factor-2 mRNA binding proteins (IGF2BPs/IMPs/VICKZs) are a family of highly conserved RBP that contains IGF2BP1, IGF2BP2, and IGF2BP3 [42,43]. IGF2BPs features two RNA recognition motif (RRM) domains and four heterogeneous nuclear ribonucleoprotein K homology (KH) domains [44]. These domains play a key role in RNA binding and the stabilization of RNA–protein complexes, which are vital for fundamental mRNA processes like transport, localization, stability, and translation. They operate by attracting target transcripts to cytoplasmic messenger ribonucleoprotein particles that gather in non-membrane-bound RNA granules, such as processing bodies and stress granules, thereby influencing mRNA decay and translation [45,46]. IGF2BP2, in particular, is integral to the development and function of the nervous system and is involved in managing cellular energy processes like lipid metabolism, glycolysis, and aerobic respiration. While IGF2BP1 and IGF2BP3 are typically minimally expressed in adult tissues, IGF2BP2’s expression is more widespread [47]. IGF2BP1 influences organ development during embryogenesis and affects brain structure and cell motility through its complex interaction with β-actin mRNA [48]. Studies in knockout mice revealed crucial roles in neocortex organization, cortical cell activity, and β-actin mRNA dynamics, illustrating IGF2BP1’s impact on cell structure and division [49].

Further research emphasizes its vital role in intestinal health and wound repair, stabilizing Occludin mRNA to maintain barrier integrity and regulating Ptgs2 mRNA in colonic stem cells for effective mucosal healing, showcasing its essential functions in both development and disease management [50,51]. However, significant re-expression of all IGF2BP genes has been observed in many human cancers, specifically CRC. The tumorigenic and metastatic roles of IGF2BP1 protein in CRC have been extensively studied, revealing their complex contributions to disease progression and treatment resistance [52,53,54,55]. For instance, LINC00858 has been shown to drive hepatic metastases in CRC by modulating miR-132–3p/IGF2BP1 signaling, enhancing the metastatic capability of CRC cells. Additionally, linc00659 interacts with IGF2BP1, stabilizing its activity on the FZD6 gene and activating the Wnt/β-catenin pathway, which is crucial for cell proliferation. Further studies have demonstrated CRC cells’ reliance on IGF2BP1 for regulating Wnt/β-catenin-responsive genes, with iCLIP analysis revealing significant changes in IGF2BP1-binding motifs in response to pathway signals [56]. Inhibition of IGF2BP1 was shown to sensitize a variety of cancer cells to therapeutics [57,58,59], and it was demonstrated to be especially effective in reducing the resistance of chemotherapy-resistant CRC cells with activated Wnt/β-catenin signaling [60].

METTL3 is a key component of the m6A methyltransferase complex, responsible for adding N6-methyladenosine (m6A) modifications to RNA, which regulates RNA stability, splicing, and translation. RNA-binding proteins (RBPs) interact with these m6A-modified RNAs to modulate gene expression, influencing processes such as cancer progression. METTL3’s involvement in m6A modification processes, such as upregulating circUHRF2 and safeguarding DDX27 protein through IGF2BP1 recruitment, underscores its role in CRC stemness and metastasis [61]. In hypoglycemic conditions or when glucose metabolism is inhibited, CRC cells become more susceptible to hyperthermia treatments. This sensitivity is linked to the upregulation of IGF2BP1 and its specific interaction with LDHA mRNA, which enhances glycolytic processes and, thus, survival under stress [62]. Furthermore, the regulatory dynamics of IGF2BP1 involving let-7 microRNA and alternative polyadenylation highlight its significant impact on CRC metastasis. The short 3’ UTR variant of IGF2BP1, which lacks microRNA binding sites and is prevalent in CRC patient samples, is associated with increased protein levels that drive proliferation and survival mechanisms vital for metastasis, particularly to the liver [63].

In conjunction with these findings, research on IGF2BP2 and IGF2BP3 has similarly elucidated their roles in CRC through m6A methylation mechanisms. IGF2BP2 enhances the stability of mRNAs like SOX2 and STAG3, promoting stemness, proliferation, and migration, whereas its high expression correlates with poor responsiveness to standard chemotherapies such as 5-fluorouracil and oxaliplatin [64]. Similarly, the overexpression of IGF2BP3 in CRC significantly correlates with poor outcomes, with its ability to stabilize mRNAs of key genes like CCND1 and VEGF enhancing cell cycle progression and angiogenesis [65]. These studies comprehensively demonstrate the pivotal roles of IGF2BP proteins in CRC pathogenesis and their potential as targets for innovative cancer therapies.

### 3.2. Inhibitors

Recent studies highlight significant advancements in cancer therapy, focusing on small-molecule inhibitors (Table 2) that target the RNA-binding proteins IGF2BPs. Mahapatra et al. developed a HTS technique to discover molecules like 6896009, which selectively curtails cancer cell proliferation by disrupting the interaction between IGF2BP1 and c-Myc mRNA, a critical regulator of this oncogene expression [66]. Similarly, another molecule, BTYNB [67], was identified for its potent inhibition of the same interaction, effectively downregulating c-Myc mRNA and protein levels, leading to decreased proliferation and growth in cancer cells, especially in ovarian and melanoma models.

Further research explored more complex interactions, such as the compound 7773, which disrupts the IGF2BP1–Kras mRNA interaction. The efficacy of 7773 in reducing Kras mRNA levels and its downstream signaling without impacting non-cancerous cells underscores its potential, especially taking into consideration the role of Kras oncogene in multiple malignancies and the difficulty of targeting active Kras proteins [68]. Building on this, the derivative AVJ16 emerged from modifications of 7773 to enhance binding efficiency and specificity, showing promising results in reducing tumor properties in cell lines expressing high IGF2BP1 levels [69].

Additionally, the research by Yang Liu et al. introduced cucurbitacin B (CuB) as a novel inhibitor targeting IGF2BP1, enhancing the immune response and reducing cancer progression in hepatocellular carcinoma by affecting m6A-modified transcripts [70]. To further optimize the therapeutic potential, A11 was developed as a derivative of CuB, which proved more potent and less toxic, indicating a promising future in clinical settings against non-small cell lung cancer (NSCLC) [71]. These studies not only demonstrated the role of IGF2BP1 in cancer progression but also emphasized the therapeutic potential of targeting this protein with small molecules to manage and treat various aggressive cancers.

Although not yet tested in CRC, the IGF2BP2 and IGF2BP3 inhibitors effectively inhibited tumor growth in model organisms and cancer cell lines. The development of small molecule inhibitors targeting IGF2BP2 and IGF2BP3 RNA-binding proteins has been reported in the literature with encouraging advances against carcinomas. The first study introduces novel inhibitors targeting IGF2BP2, which is linked to poor prognosis in CRC and liver cancer. Utilizing gene expression analysis and various experimental models, the study confirms IGF2BP2’s role in tumorigenesis and identifies two classes of compounds, benzamidobenzoic acid and ureidothiophene, which effectively reduce tumor growth in zebrafish embryos [72]. Another paper explores targeting IGF2BP2 in T-cell acute lymphoblastic leukemia (T-ALL), revealing its role in promoting cell proliferation and impairing chemotherapy sensitivity by stabilizing NOTCH1 mRNA. The inhibitor JX5 shows promise in reducing T-ALL proliferation and enhancing chemosensitivity, underscoring the potential of IGF2BP2 as a therapeutic target in leukemia [73]. Additionally, research into IGF2BP3’s role in ovarian cancer (OC) identifies the small molecule AE-848, which disrupts IGF2BP3’s interaction with target mRNAs, leading to decreased levels of factors like c-MYC, VEGF, and CDK2. AE-848 inhibits OC cell proliferation and invasion in vitro and demonstrates significant anti-tumor effects in vivo, highlighting its potential as a targeted therapy for OC. Given the role of IGF2BP2 and IGF2BP3 in CRC, these inhibitors may be potentially effective, although they still need to be tested experimentally in preclinical models of CRC. The interaction of inhibitors with IGF2BPs was comprehensively reviewed by Cai et al. elsewhere [74].

## 4. Musashi

### 4.1. (Patho)physiological Function

Musashi proteins, specifically Musashi-1 (MSI1) and Musashi-2 (MSI2), are essential RNA-binding proteins that play pivotal roles in stem and progenitor cell regulation across various biological systems [75]. MSI1 is primarily expressed in the nervous system, particularly in neural stem cells. It influences crucial processes such as neural differentiation and stem cell pluripotency [76,77]. It achieves this by interacting with other regulatory proteins like LIN28 and PABP with its C-terminal end [78]. This interaction influences the miRNA activities and mRNA stability crucial for stem cell regulation.

On the other hand, MSI2 is predominantly involved in the self-renewal and proliferation of hematopoietic stem cells [79]. Both proteins share a substantial degree of structural similarity and functionally contribute to the post-transcriptional regulation of gene expression, impacting essential cellular processes like cell differentiation and organ development [80]. Studies across species, from Drosophila to humans, have underscored the evolutionary conservation of Musashi proteins’ roles [81]. Its role is significant in maintaining the integrity of stem cell functions and has potential implications in various diseases, including cancer and developmental disorders.

MSI2 was demonstrated as essential for the survival of colorectal cancer cells but not for non-small cell lung cancer cells. Through MSI2 knockout models, 38 potential MSI2-targeted genes were identified, significantly enhancing the understanding of MSI2’s role in cancer biology [82]. In CRC, Musashi-1 (MSI1) plays a role in enhancing chemoresistance and enhancing the development of CD44+ colorectal cancer stem cells (CSCs). Specifically, MSI1 promotes the development of CD44+ colorectal CSCs and the formation of anti-apoptotic stress granules during 5-fluorouracil treatment, contributing significantly to treatment resistance. The presence of MSI1 in early-stage CRC (stages IIA and IIB) highlights its role in both cancer progression and resistance to chemotherapy [83]. MSI2 shows increased expression from colon polyps to CRC and is associated with poor clinical outcomes. The study, involving 125 patients, demonstrates that elevated MSI2 levels correlate with reduced progression-free and overall survival, higher tumor grades, and right-sided tumor localization. In CRC cells, the knockdown of MSI2 reduced cell proliferation, survival, and clonogenic capacity and modulated key markers like TGFβ1, E-cadherin, and ZO1 [84]. MSI2 regulates ferroptosis, a programmed cell death driven by iron and lipid peroxide accumulation. MSI2 is upregulated in CRC and correlates with high levels of ferroptosis inhibitors, suggesting MSI2’s involvement in cancer cell survival and malignancy. Knocking down MSI2 in CRC cells reduced their proliferation, migration, invasion, enhanced ferroptosis, indicated by increased intracellular iron, reactive oxygen species, and lipid peroxidation. This effect was mechanistically linked to MSI2’s interaction with the MAPK signaling pathway, mainly by inhibiting HSPB1 phosphorylation [85]. These findings suggest that targeting MSI1 and MSI2 could be a promising strategy to improve treatment responses and curb metastasis in CRC, underscoring its potential as a therapeutic target.

### 4.2. Inhibitors

In an attempt to discover an inhibitor for MSI (Table 3) in one of the first studies, a library of 6208 compounds was screened using an FPA, which identified 27 molecules inhibiting MSI with varying specificity and potency. A pilot screen was performed against both MSI1 and MSI2, identifying 7 molecules for MSI1, 15 for MSI2, and 5 that inhibited both. A secondary FP dose–response screen validated 3 MSI inhibitors with IC_50_ below 10 μM [86]. The inhibitors specifically targeted the MSI family of RNA-binding proteins. The same research group later, in a separate study, identified and characterized the small molecule Ro 08-2750 (Ro) as an inhibitor targeting the Musashi (MSI) family of RBPs, particularly MSI2, in the context of acute myeloid leukemia (AML). Ro was shown to bind directly and selectively to MSI2, competing with its RNA binding activity in biochemical assays. This binding led to increased differentiation and apoptosis in both mouse and human myeloid leukemia cells, along with the inhibition of known MSI targets and alteration of global gene expression patterns. Gene-set enrichment analysis showed a substantial overlap of functional pathways, particularly involving c-MYC and leukemia-associated gene sets, demonstrating Ro treatment’s capacity to mimic the MSI2-related gene expression patterns. The study revealed an IC_50_ of 2.7 ± 0.4 μM for Ro, validating its efficacy in inhibiting MSI2’s RNA-binding activity [87].

A separate study identified palmatine as a potent MSI2 inhibitor, showing specificity in binding MSI2 over other proteins. Palmatine was particularly effective in inhibiting MSI2-dependent growth of CRC cells, with direct binding confirmed through various assays [82]. Another study focuses on designing small-molecule inhibitors targeting specifically MSI1 and MSI2. Using a novel “hotspot mimicry” approach, potential inhibitors were developed by mimicking the critical interaction points within the protein–RNA complex. A high-throughput screening of approximately 7 million compounds identified a potent inhibitor, R12-8-44-3, which effectively disrupts the MSI-RNA interaction. R12-8-44-3 showed a notable IC_50_ value of 9.7 µM, proving its efficacy in inhibiting MSI1 and MSI2’s RNA-binding capabilities and presenting a promising approach for targeting RBPs in therapeutic applications [88].

**Figure 2:**
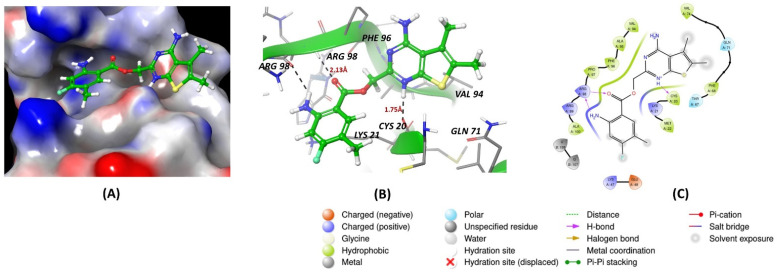

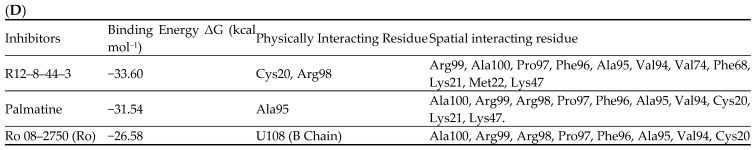
(**A**,**B**) show 3D representations of the binding and surface view of inhibitor R12–8–44–3 with RNA binding protein Musash1 (PDBID: 2RS2). (**C**) shows 2D representations of binding interactions of Musashi1. (**D**) The binding energy of inhibitors with RNA binding protein Musashi 1 along with interacting residues. Color code: green = carbon, white = hydrogen, blue = nitrogen, and red = oxygen.

Docking studies revealed that the inhibitor R12–8–44–3 exhibited the highest binding energy among the Musashi1 inhibitors (Figure 2D). Figure 2A–C indicates that the inhibitor interacts solely with the protein, without engaging with RNA. It forms a hydrogen bond with protein residue Arginine 898 through its benzene amino group and ester carbonyl group. Additionally, the heterocyclic portion of the inhibitor establishes a hydrogen bond with Cysteine 20 via its nitrogen atom, contributing to its strong protein binding.

**Figure 3:**
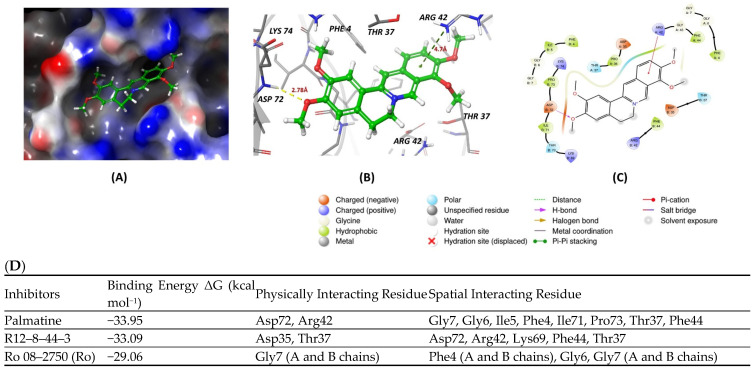
(**A**,**B**) show 3D representations of the binding and surface view of inhibitor palmatine with RNA binding protein Musash2 (PDBID: 6DBP). (**C**) shows 2D representations of binding interactions of Musashi2. (**D**) The binding energy of inhibitors with RNA binding protein Musashi 2 along with interacting residues. Color code: green = carbon, white = hydrogen, blue = nitrogen, and red = oxygen.

All inhibitors were further assessed for their interactions with the MS2 protein. Among them, palmatine demonstrated a slightly better energy score compared to the other inhibitors (Figure 3D). It formed a hydrogen bond with Aspartate 72 of MS2, with a bond length of 2.78 Å, and engaged in a pi–cation interaction with Arginine 42, with a bond length of 4.7 Å. These interactions contributed to its relatively stronger binding affinity with the protein (Figure 3A–C).

## 5. HuR

### 5.1. (Patho)physiological Function

Human antigen R (HuR) is a crucial RBP from the embryonic lethal abnormal visual (ELAV) family, recognized for its widespread expression in various tumors, unlike its neuron-specific counterparts HuB, HuC, and HuD [89,90]. Initially identified in patients with paraneoplastic neurological symptoms, HuR has expanded its known roles to include pivotal functions in cancer progression [91]. It is involved in angiogenesis, apoptosis, invasion, metastasis, and resistance to chemotherapy and radiotherapy, making it a promising target for therapeutic intervention and a biomarker for prognosis in oncology [92]. At the molecular level, HuR is encoded on chromosome 19 and primarily localized in the nucleus in resting cells, relocating to the cytoplasm in response to various stimuli where it stabilizes and regulates the translation of mRNAs containing adenosine/uridine-rich elements. This post-transcriptional regulation affects a range of critical proteins, including oncogenes, growth factors, and anti-apoptotic factors, highlighting HuR’s role in promoting malignant behaviors and impacting patient outcomes. HuR also plays an important role in the normal growth of small intestine mucosa by modulating Wnt signaling pathways through the upregulation of LRP6 expression [93].

Research from early 2000 demonstrated that HuR promotes the proliferation of CRC RKO cells by targeting cyclins A and B1. The HuR small-molecule inhibitor MS-444 has been shown to inhibit tumor cell growth and induce apoptosis in CRC cells [94]. Notably, MS-444 also curtailed the growth of CRC xenograft tumors through enhanced apoptosis and reduced angiogenesis following its intraperitoneal administration. Moreover, the cytoplasmic presence of HuR in CRC is significantly linked with elevated COX-2 expression and advanced tumor stages. HuR enhances COX-2 translation by binding to its mRNA 3′-UTR, while MS-444 blocks HuR’s nuclear transport, suppressing COX-2 expression and exerting an anti-tumor effect [94,95].

Furthermore, HuR’s regulation involves various factors, including miR-155-5p, which targets the HuR mRNA 3′-UTR and reduces HuR expression and migration in colon cancer HT-29 cells [96]. Protein kinase Cδ phosphorylation affects HuR’s functionality and expression, with more pronounced phosphorylation in CRC DLD-1 cells compared to normal colonic epithelial CCD 841 cells. Phosphorylated HuR significantly enhances the migration and proliferation of these cells. The tumor suppressor miR-22 targets HuR to inhibit CRC cell proliferation and migration and diminish colorectal xenograft tumor growth [97]. Elevated HuR expression correlates with increased malignancy and poorer survival rates in CRC patients. A study analyzing 560 CRC patient specimens revealed that high HuR levels are associated with advanced disease and predict poorer patient outcomes [98]. This highlights HuR’s integral role in CRC biology and underscores the importance of further investigating its contribution to tumor development for better prevention and treatment strategies.

### 5.2. Inhibitors

Among all known HuR inhibitors to date (Table 4), the first were identified and characterized in 2007: dehydromutactin, MS-444, and okicenone were derived from microbial broths (*Actinomyces* sp.) and found to effectively interfere with HuR’s RNA binding, affecting HuR trafficking and cytokine expression, which influences T-cell activation. The compounds appear to inhibit the formation of HuR dimers before RNA binding, highlighting their potential as valuable tools for studying HuR function and the possibility of targeting HuR as a therapeutic approach in treating cancer. The study utilizes a combination of mathematical and experimental analysis to explore the inhibitors’ mode of action, providing a promising foundation for further research into HuR’s role in malignant processes [99]. A separate study to identify and validate novel small molecule disruptors of the HuR–mRNA interaction focused on compounds that competitively bind to HuR to inhibit its function. A high-throughput screening of approximately 6000 compounds using an FPA identified a cluster of potential disruptors. Subsequent validation determined that these compounds effectively disrupted the HuR–ARE interaction at nanomolar levels. MS-444 emerged as a potent inhibitor, inhibiting HuR homodimerization, thereby disrupting the stabilizing interaction of HuR with target mRNAs [100]. Another study focused on identifying small-molecule inhibitors that target the HuR/RNA interaction. This is essential for cancer development because it stabilizes mRNAs associated with proto-oncogenes and other growth factors. Using a combination of FPA and NMR validation, NCI diversity set V library was screened, identifying several potential inhibitors. Notably, compounds C10 and C11 were highlighted for their specific interactions with HuR: C10 interfered with HuR/RNA binding, while C11 disrupted HuR oligomerization, both critical mechanisms for the post-transcriptional regulation of gene expression [101].

CMLD-2 was identified through HTS of 6000 compounds and showed potent activity against HuR by disrupting its interaction with target mRNA. In non-small cell lung cancer (NSCLC) cell lines, CMLD-2 induced dose-dependent cytotoxic effects, causing cell cycle arrest in the G1 phase and triggering apoptosis. The study demonstrated that CMLD-2 specifically reduced the mRNA and protein levels of HuR-regulated genes such as Bcl2 and p27, enhancing pro-apoptotic pathways while showing minimal effects on normal human fibroblasts. Notably, CMLD-2 also induced mitochondrial perturbations and activated caspase-9 and -3, leading to PARP cleavage in tumor cells, with an observed IC_50_ of 28.9 µM for CRC cells and 18.2 µM for pancreatic cancer cells [102]. KH-3 is another potent HuR inhibitor, which significantly suppresses breast cancer invasion and metastasis by disrupting the HuR–FOXQ1 mRNA interaction. KH-3, by inhibiting this interaction, has shown promising results in both in vitro and in vivo models, enhancing survival and reducing tumor metastasis, thus positioning it as a potential therapeutic candidate for combating metastatic breast cancer [103]. Another inhibitor dihydrotanshinone-I (DHTS) was found to disrupt the RNA-binding capability of HuR by forcing the protein into a closed conformation, thereby blocking RNA binding. This mechanism is facilitated through DHTS’s attachment to the same HuR sites as RNA. NMR titration and molecular dynamics simulations pinpointed the specific residues in HuR’s RNA-recognition motifs that DHTS affects. Furthermore, DHTS was shown to selectively block HuR’s interaction with weaker mRNA targets and enhances its association with mRNAs, having longer 3′UTRs and a higher density of U/AU-rich elements. In vivo studies reinforced DHTS’s viability as an anticancer agent by substantially reducing tumor growth in HuR-dependent xenograft models without causing systemic toxicity. Motivated by DHTS’s effectiveness, the researchers developed a series of tanshinone analogs, among which compounds 6a and 6n were notably more potent and effective at disrupting HuR–RNA interactions [104,105].

**Table 4:** HuR inhibitors.

Structure	Name	IC_50_ (µM)	Reference
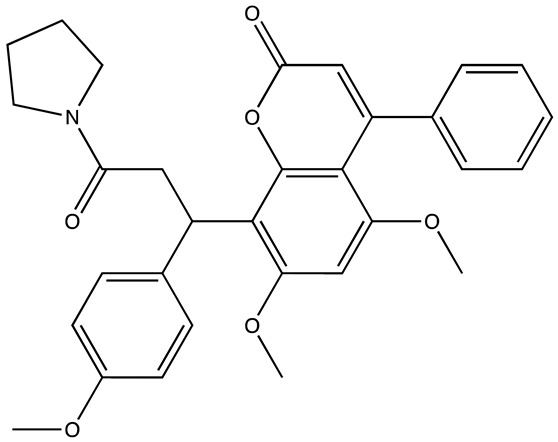	CMLD2	2.4	[100]
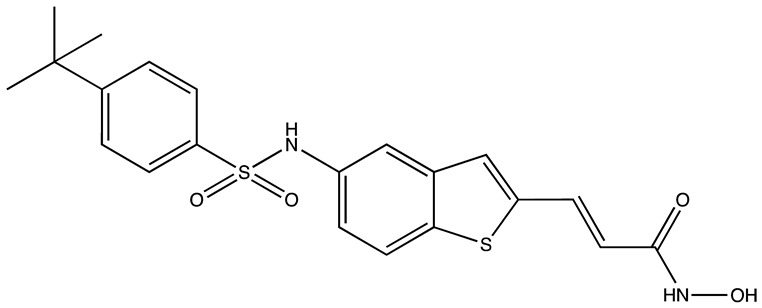	KH-3	3.5	[103]
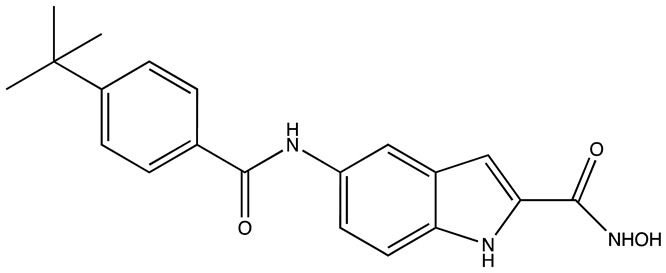	1C	4.74	[106]
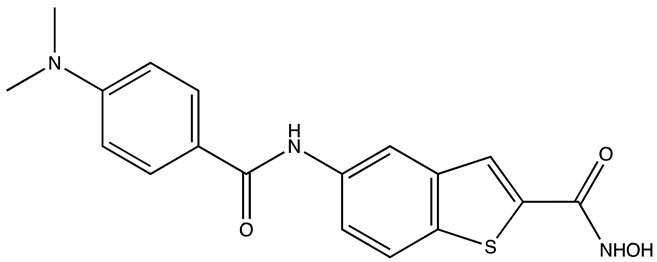	7C	1.31	[106]
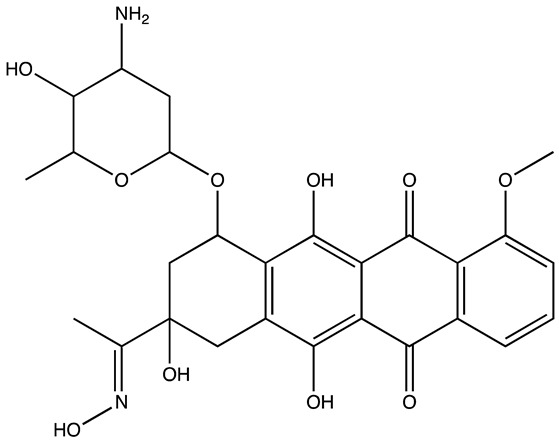	C10	21.7	[101]
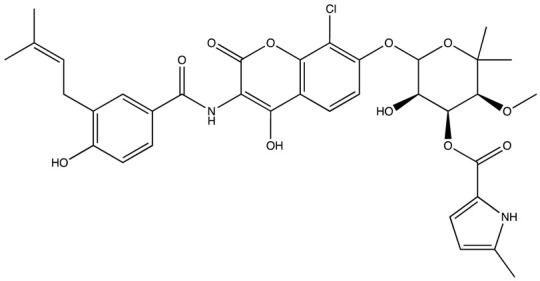	C11	41.9	[101]
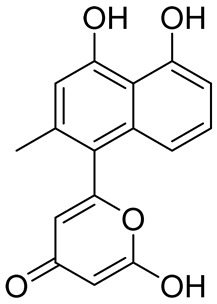	Dehydromutactin (1)	0.19	[99]
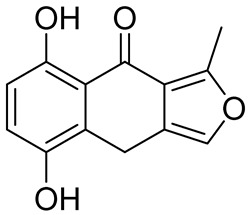	MS-444	0.04	[99]
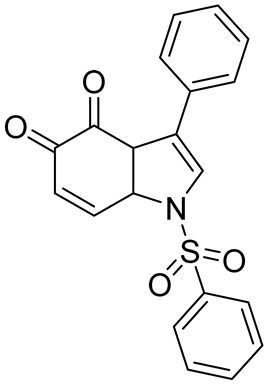	6a	0.0128	[105]

Through a hybrid approach combining fragment analysis and structural optimization, researchers developed 36 new compounds. The two most promising inhibitors identified were 1c and 7c. These inhibitors function by directly binding to HuR’s RNA-binding pocket, effectively inhibiting the growth of cancer cells reliant on HuR and suppressing cancer cell invasion. Specifically, inhibitor 1c significantly inhibited tumor growth when administered intraperitoneally and displayed a synergistic effect when combined with chemotherapy (docetaxel) in breast cancer xenograft models. It works by interfering with the HuR−TGFB/THBS1 axis [106]. Another novel approach for targeting HuR by utilizing a single-domain antibody (VHH) with low nanomolar affinity to HuR, researchers developed a TRIM21-based biological proteolysis-targeting chimera (bioPROTAC) capable of degrading endogenous HuR. This degradation demonstrated a significant reversal of tumor-promoting properties in cancer cells in vivo, highlighting the therapeutic potential of this strategy. The bioPROTAC, specifically targeting HuR, is a promising alternative to small-molecule inhibitors [107].

**Figure 4:**
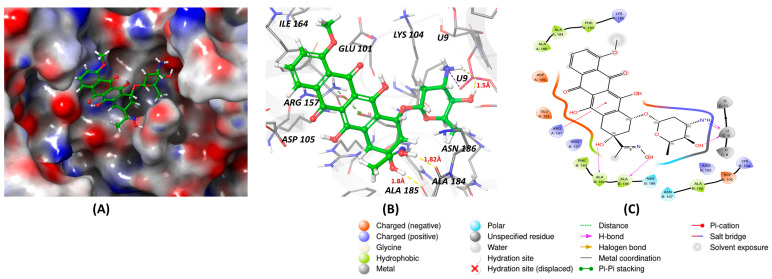

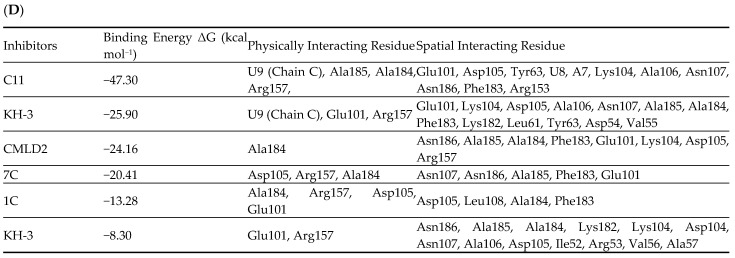
(**A**,**B**) show 3D representations of the binding and surface view of inhibitor C11 with RNA binding protein HUR (PDBID: 4ED5). (**C**) shows 2D representations of binding interactions of HUR. (**D**) The binding energy of inhibitors with RNA binding protein HUR along with interacting residues. Color code: green = carbon, white = hydrogen, blue = nitrogen, and red = oxygen.

All known HuR inhibitors were docked to the human HuR protein using PDB ID: 4ED5 to check the binding affinity. Inhibitor C11 demonstrated strong binding affinity with both the protein and RNA (Figure 4A–C). It formed hydrogen bonds through its hydroxy groups with protein residues Ala185, Ala184, and with the U9 residue of the C chain in the RNA. Additionally, one of the aromatic rings of C11 exhibited a pi–cation interaction with Arg157 of the protein. The protein surface diagram further revealed that inhibitor C11 occupied the binding pocket of the protein. The inhibitor also showed spatial interactions with the protein residues listed in Figure 4D, contributing to its higher binding affinity compared to other inhibitors.

## 6. CELF1

### 6.1. (Patho)physiological Function

CELF1, a member of the CELF protein family, plays a critical role across various biological and disease processes by binding to GU-rich elements within mRNAs to modulate activities such as mRNA splicing, translation, and attenuation [108]. This RBP maintains body homeostasis, influencing embryonic and cardiac development, bone and adipose tissue differentiation, and germ cell formation. Structurally, CELF1 comprises three RNA recognition motifs (RRMs)—RRM1 and RRM2 are closely located at the N-terminal, whereas RRM3 is near the C-terminal, with a significant junction region between RRM2 and RRM3 [109]. Crucially, CELF1 is essential for gametogenesis and embryonic development, as demonstrated in various animal models like mice, Xenopus, and zebrafish, where its deletion leads to reproductive deficiencies. In mice, CELF1 is also noted for its widespread expression in the small intestinal epithelium, where it is subject to regulation by mir-503 and is associated with P bodies, highlighting its complex regulatory capacity at the post-transcriptional level [110]. CELF1 protein levels were demonstrated to be increased in CRC tissues and cell lines, and its elevated expression is correlated with the incidence of liver metastasis. CELF1 was also shown to promote the growth and spread of CRC cells and to be highly expressed in liver metastatic lesions [111].

Additionally, CELF1 is known to boost the migratory and invasive abilities of CRC cells and their resistance to chemotherapy by interacting with ETS2 mRNA, which results in heightened ETS2 expression. Functionally, overexpression of CELF1 was shown to enhance CRC cell proliferation, migration, invasion, and resistance to oxaliplatin (L-OHP), a joint chemotherapeutic agent. Conversely, knocking down CELF1 improved CRC cell sensitivity to L-OHP. Similar effects were observed with ETS2 overexpression, which also promoted malignant behaviors and chemoresistance. Significantly, L-OHP resistance driven by CELF1 overexpression could be reversed by knocking down ETS2, suggesting that CELF1 upregulates ETS2 by binding to its 3′-UTR. These findings underscore CELF1’s pivotal role in CRC tumorigenesis and chemoresistance, highlighting its potential as a therapeutic target to counteract chemoresistance in CRC [112].

### 6.2. Inhibitor

Compound 27 (Table 5) was shown to effectively disrupts CELF1’s interaction with RNA, thereby inhibiting the activation of hepatic stellate cells (HSCs)—a key event in the progression of liver fibrosis. This inhibition subsequently leads to a reduction in liver fibrosis symptoms in mouse models. Compound 27 works by directly competing with RNA to bind to CELF1, thereby modulating the stability and decay of mRNAs involved in fibrotic processes, such as IFN-γ mRNA. A derivative of Compound 27, Compound 841, was demonstrated to exhibits enhanced selectivity and potency in inhibiting CELF1. This compound represents a significant advancement in the pharmacological targeting of this RBP, providing a promising new avenue for treating liver fibrosis and potentially other related diseases where CELF1 plays a detrimental role. The findings open new possibilities for using RNA-binding protein inhibitors in disease modification and treatment, particularly in conditions characterized by excessive tissue scarring and fibrosis [113].

**Figure 5:**
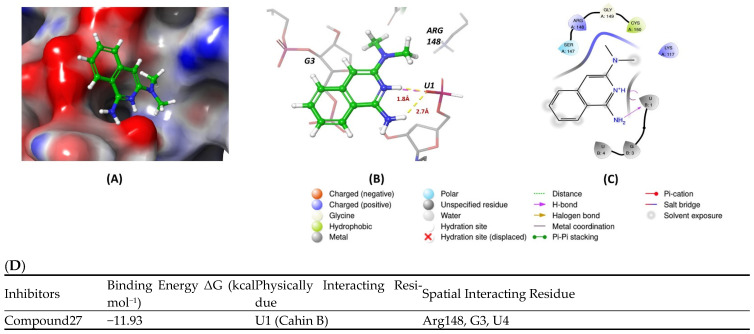
(**A**,**B**) show 3D representations of the binding and surface view of inhibitor compound27 with RNA binding protein CELFI (PDBID: 3NMR). (**C**) shows 2D representations of binding interactions of CELFI. (**D**) The binding energy of inhibitors with RNA binding protein CELFI along with interacting residues. Color code: green = carbon, white = hydrogen, blue = nitrogen, and red = oxygen.

Molecular docking was conducted using PDB ID 3NMR, following the previously described methods. The ligand did not interact directly with protein residues (Figure 5D). However, it formed two hydrogen bonds via its pyridinium nitrogen and dialkyl nitrogen atoms with the U1 residue of the RNA. Additionally, the ring nitrogen of the ligand participated in a salt bridge interaction with the U1 residue, further stabilizing the binding with the RNA (Figure 5A–C).

## 7. Molecular Docking Methods

The molecular docking procedures were carried out using the Maestro software platform (version 13.2.128; Schrödinger LLC, New York, NY, USA). To prepare the RNA binding proteins (PDB codes: 5UDZ, 4ED5, 2RS2, 6DBP, 3NMR), the Protein Preparation Wizard was used, which involved removing water molecules and adding bond orders and hydrogen atoms to the protein structures. Molecular configurations of the compounds were created using the LigPrep module with the OPLS3e force field, allowing the exploration of various tautomeric and ionization states, leading to the generation of 3D molecular structures. The protein binding sites were identified using Maestro’s Sitemap module, and the top-ranked site was selected to generate a receptor grid [114]. Ligand docking was performed using the Glide module, applying its default parameters, while the Prime module was used to compute the binding free energies [115,116].

## 8. Conclusions

RBPs are important regulators of gene expression at the post-transcriptional level, affecting processes such as mRNA splicing, stability, and translation. Their misregulation has been linked with various diseases, including cancer. In CRC, RBPs have attracted attention due to their significant roles in promoting tumor growth, metastasis, and therapy resistance, particularly through interactions with oncogenic mRNA and their maintenance of cancer cell properties. Targeting RBPs for therapeutic purposes has shown great promise, as evidenced by recent studies demonstrating their potential as novel cancer treatment targets. Inhibitors of specific RBPs, such as LIN28, IGF2BPs, HuR, and Musashi, have shown efficacy in decreasing tumor growth, enhancing the differentiation of cancer cells, and reducing resistance to chemotherapy. Therapeutic strategies using small molecule inhibitors have demonstrated success in preclinical models by disrupting the interactions between RBPs and their RNA targets, thereby inhibiting tumor progression and sensitizing tumors to treatment. Given that many RBPs are also vital for the survival of normal cells, selective targeting is critical. Furthermore, more research is needed to understand the precise cancer-specific functions of RBPs, as some can behave as oncogenes in one context and as tumor suppressors in another. Applying cutting-edge techniques such as eCLIP will be pivotal in identifying specific RNA targets of RBPs in cancer. This information could help in the development of more selective inhibitors that disrupt only the cancer-related functions of RBPs.

Despite these advances, significant challenges remain. One possible way to improve RBP targeting is to design specific degraders, such as PROTACs, that employs ubiquitin ligases to selectively degrade target RBPs, effectively disrupting protein–RNA interactions (PRIs). This technique utilizes small molecules as “molecular glue” to stabilize interactions between an E3 ubiquitin ligase and its non-native substrates, facilitating their ubiquitination and subsequent degradation, potentially offering superior efficacy compared to traditional inhibitors. This approach not only addresses the limitations inherent in traditional inhibitors but also broadens the spectrum of “undruggable” proteins that can be targeted, paving the way for novel therapeutic developments. Another exciting possibility is to utilize novel in silico methodology, such as the molecular docking presented here, to both improve selectivity and efficacy of existing RBP inhibitors and to design novel potent compounds. Another critical issue is improving delivery methods for targeting RBPs, specifically within cancer cells, while minimizing effects on normal tissues, that is being addressed by improving the bioavailability of existing inhibitors and by a variety of tumor-specific targeting approaches.

In conclusion, RBPs offer a promising target for the treatment of colorectal cancer, particularly in overcoming therapy resistance and enhancing the efficacy of existing treatments. Continued research into more effective delivery systems and selective inhibitors will be essential in realizing the full therapeutic potential of these proteins in cancer treatment.

## Figures and Tables

**Table 2:** IGF2BP inhibitors.

Structure	Name	K_D_	Reference
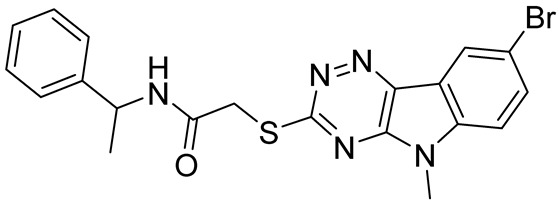	6896009	Not known	[66]
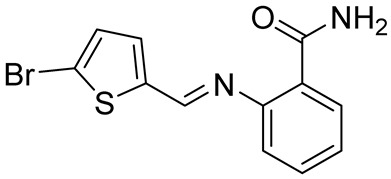	BTYNB	Not Known	[67]
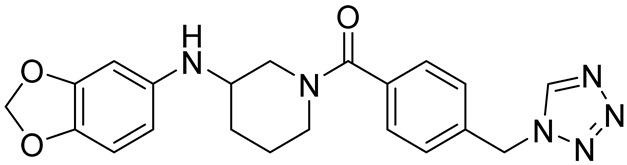	7773	17 µM	[68]
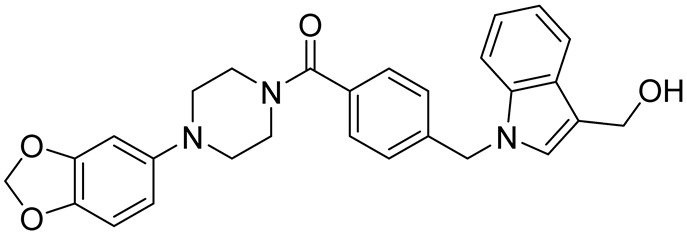	AVJ16	1.4 µM	[69]
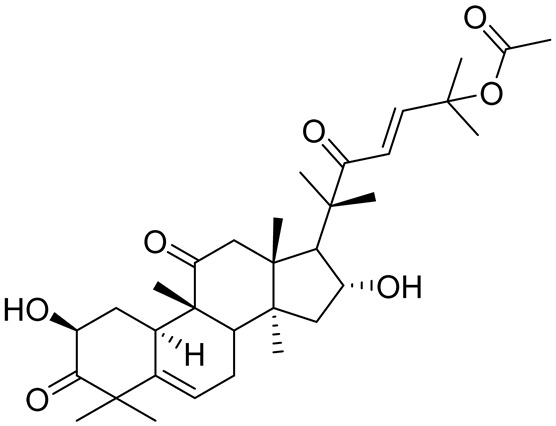	CuB	1.2 µM	[70]
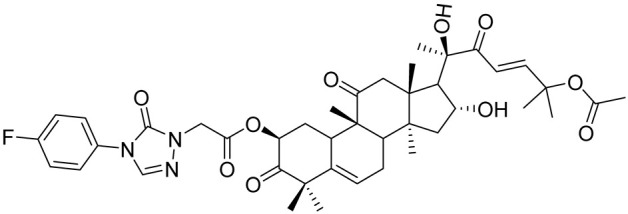	A11	2.88 nM	[71]

**Table 3:** Musashi inhibitors.

Structure	Name	IC_50_ (µM)	Reference
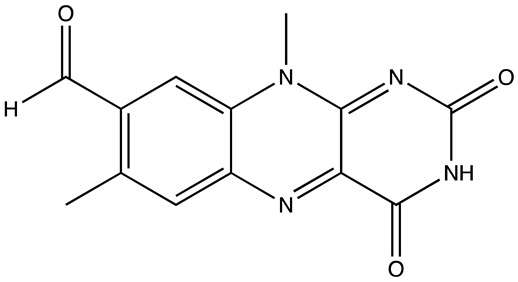	Ro 08–2750 (Ro)	2.7	[87]
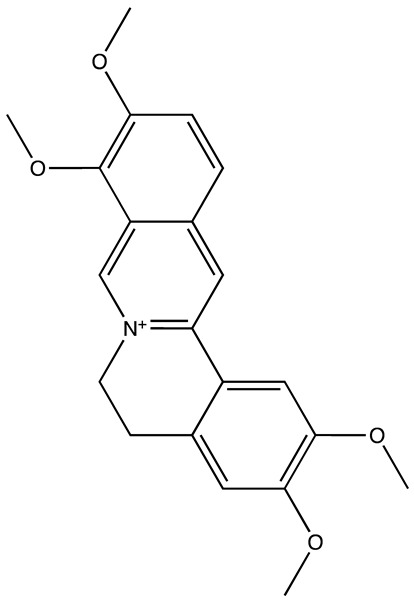	Palmatine	N/A	[82]
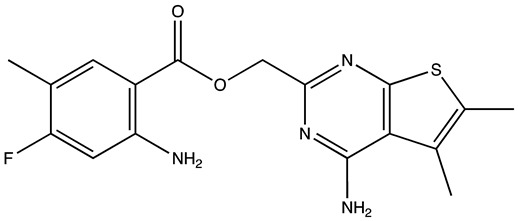	R12–8–44–3	9.7	[88]

**Table 5:** CELF1 inhibitor.

Structure	Name	IC_50_ (µM)	Reference
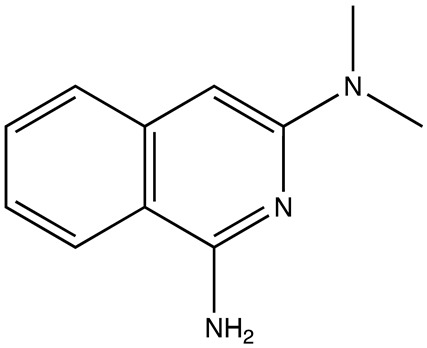	Compound 27	22.99	[113]

## Data Availability

There are no new data generated in the review.
